# Genetic Engineering Efforts to Generate Chlamydomonas Strains for Large‐Scale Biomolecule Production

**DOI:** 10.1111/1751-7915.70466

**Published:** 2026-11-03

**Authors:** Sofia Inturri, Nicolas Rouhier, Claire Remacle

**Affiliations:** ^1^ Genetics and Physiology of Microalgae, InBioS/Phytosystems Research Unit University of Liège Liège Belgium; ^2^ Université de Lorraine, INRAE, IAM Nancy France; ^3^ Institut Universitaire de France Paris France

**Keywords:** chloroplast, genetic engineering, genetic tools, industrial biotechnology, nucleus, synthetic biology chassis, unicellular green microalga

## Abstract

*Chlamydomonas reinhardtii* is an established model microalga. Its easy manipulation, metabolic flexibility and tractable genomes allowed researchers to investigate fundamental processes such as photosynthesis using genetic studies for decades. Chlamydomonas is not naturally rich in lipids or pigments compared to industrially relevant microalgae. However, we highlight how its biotechnological potential relies on advanced genetic and metabolic engineering strategies, now possible due to the availability of extensive toolbox and synthetic biology platforms, which can improve industrial traits and allow to produce a variety of biomolecules of biotechnological interest. We provide examples of biomolecule production (terpenoids and recombinant proteins), and show that productivities (mg/L) are in the same range or could even be higher than those reached in other photosynthetic chassis (e.g., cyanobacteria or mosses). We point out that despite these achievements, the industrial implementation of *C. reinhardtii* is still lacking. Further technological advancements as well as clear international regulations and social acceptance are fundamental to reach the full potential of this microalga.

## Graphical abstract

This represents the main aspects relevant to potential industrialization of Chlamydomonas.

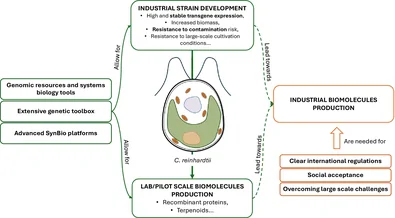

## 1 Introduction

The original description of *Chlamydomonas reinhardtii* was made in Europe in the late 19th century, but most of the isolates are found in North America, and a few in Japan (Craig et al. 2019). Strains and mutants used for the genetic and biochemical analyses are derived from meiotic products of a single zygote isolated in a potato field of Massachusetts in the United States in 1945 (Craig et al. 2019).

The ability of *C. reinhardtii* to grow in phototrophic (light), mixotrophic (light + acetate) and heterotrophic (dark + acetate) conditions explains why photosynthetic (acetate‐dependent) and respiratory‐deficient (light‐dependent) mutants could be isolated early in 60s and 70s (reviewed in Salomé and Merchant 2019). Metabolic mutants could also be scored, including auxotrophic mutants for arginine (Matagne 1978) and mutants unable to grow on nitrate (Fernandez and Matagne 1984).

In 90s, vectors containing the argininosuccinate lyase (*ARG7*) and nitrate reductase (*NIA1*) genes were used for transformation of the arginine‐ and nitrate‐deficient mutants cited above (Debuchy et al. 1989; Kindle 1990). Since these pioneer works, dominant markers conferring resistance to several antibiotics and allowing direct transformation of wild‐type Chlamydomonas cells were used (reviewed in Perozeni and Baier 2023). Successful transformation of the chloroplast genome was also achieved (Boynton et al. 1988).

*C. reinhardtii* genome was one of the first eukaryotic genomes to be sequenced (Merchant et al. 2007). Several versions of the genome have been published since then, using different strains. The latest version was released in 2023 for the strain CC‐4532 (Craig et al. 2023). The genome contains 17 chromosomes for a total length of 114 Mb and is 64% GC‐rich. The average number of introns per gene is 6.4 (Merchant et al. 2007), compared to four introns per gene in *Arabidopsis thaliana* (Reddy 2007) and alternative splicing is estimated to occur in 20% of the genes (Raj‐Kumar et al. 2017), also much higher than in *A. thaliana* (12%) (Labadorf et al. 2010). This indicates that regulation of mRNA expression is relatively complex in *C. reinhardtii* , an important factor when designing transgenes (see below).

*C. reinhardtii* is a well‐established model organism in many research fields, including photosynthesis, organelle biology, cilia structure and function (reviewed in Salomé and Merchant 2019). It is not particularly enriched in proteins, pigments or lipids, compared to microalgae such as *Chlorella* sp., *Haematococcus pluvialis* or *Nannochloropsis* sp., which are cultivated at industrial scale for the biotechnological production of these molecules (Araújo et al. 2021). However, Chlamydomonas is the most well characterized unicellular microalga and the one with the most developed genetic toolkits and resources, both for the nuclear and chloroplast genomes (see Table 1). The microalga presents established methods for transformation, different strategies for genetic manipulation and transformants selection, as well as standardized Modular Cloning (MoClo) toolkits and recently developed synthetic biology (SynBio) platforms. This allows the generation of highly engineered strains capable of producing a wide variety of biomolecules and heterologous proteins, as will be demonstrated below. Additionally, at this time, it presents the most advanced microalgal Genome‐scale Metabolic Models (GEMs), which will be crucial in helping in the identification of productivity bottlenecks and guiding metabolic engineering at system‐levels (Tamburro and Boyle 2025). Thus, *C. reinhardtii* represents a promising photosynthetic chassis for a more sustainable biomanufacturing, based preferentially on CO_2_ and light for production of compounds.

**TABLE 1:** Comparison of the genetic toolbox, resources and SynBio platforms available for nuclear and chloroplast engineering in model and industrially relevant microalgae. The table was adapted from table 2 of Kumar et al. 2020, updated and expanded for the selected microalgae (Kumar et al. 2020).

Organism	Compartment	Transformation method	Genetic manipulation	Selectable markers	Resources, tools, SynBio platforms	References
*Chlamydomonas reinhardtii*	Nucleus	Glass‐beads: Established, Electroporation: Established	Gene expression, Genome editing, RNAi	Auxotrophy rescue, Herbicide resistance, Antibiotic resistance: Multiple	CRC, CLiP library, pcGEM, MoClo, Biofoundry	Salomé and Merchant 2019; Tamburro and Boyle 2025; Douchi et al. 2026
	Chloroplast	Biolistic: Established	Gene expression, Gene disruption	Auxotrophy rescue, Herbicide resistance, Antibiotic resistance: Multiple	MoClo, GEM	Esland et al. 2018; Bjerkelund Røkke et al. 2020; Cutolo et al. 2022; Inckemann et al. 2025
*Phaeodactylum tricornutum*	Nucleus	Biolistic: Established, Electroporation and Bacterial conjugation: Achieved	Gene expression, Genome editing, RNAi	Auxotrophy rescue, Herbicide resistance, Antibiotic resistance	DiatOmicBase, MoClo, GEM	Russo et al. 2023; Tamburro and Boyle 2025; Walker et al. 2026
	Chloroplast	Electroporation: Achieved	Gene expression	Antibiotic resistance (Chloramphenicol^R^)	GEM	Bjerkelund Røkke et al. 2020; Cutolo et al. 2022
*Nannochloropsis oceanica*	Nucleus	Electroporation	Gene expression, Genome editing, RNAi	Antibiotic resistance	NanDeSyn, CRISPRi tool, MoClo	Poliner et al. 2018; Gong et al. 2020; Naduthodi et al. 2021; Guo et al. 2026
	Chloroplast	Electroporation: Achieved	Gene expression	Antibiotic resistance (Zeocyn^R^)	GEM	Bjerkelund Røkke et al. 2020; Cutolo et al. 2022
*Chlorella vulgaris*	Nucleus	Electroporation, glass‐bead agitation, and agrobacterium‐mediated	Gene expression	Antibiotic resistance	GEM	Yang et al. 2016; Tamburro and Boyle 2025
	Chloroplast	Biolistic: Achieved	Gene expression	Antibiotic resistance (Spectinomycin^R^)	N/A	Cutolo et al. 2022
*Haematococcus pluvialis*	Nucleus	Biolistic: Achieved	Gene expression	Antibiotic resistance (Spectinomycin^R^)	GEM	Yuan et al. 2019; Tamburro and Boyle 2025
	Chloroplast	Biolistic: Achieved	Gene expression	Herbicide resistance (Bialaphos^R^), Antibiotic resistance (Spectinomycin^R^)	N/A	Cutolo et al. 2022

Abbreviations: (pc)GEM, (protein constrained) Genome Scale Metabolic Model; CLiP, Chlamydomonas Library Project; CRC, Chlamydomonas Resource Center; CRISPRi, CRISPR interference; MoClo, modular cloning; N/A, not applicable; RNAi, RNA interference.

## 2 Genome Engineering—Limitations and Recent Advancements

*C. reinhardtii* 's most valuable compartments for manipulation towards biotechnological exploitation are the nucleus and the chloroplast. Both have specific advantages and limitations, which are being highlighted by recent studies and technological advancements.

### 2.1 Nuclear Engineering

For many years, research was limited by the poor expression of nuclear transgenes. The low transgene expression is caused by epigenetic mechanisms. Exogenous DNA is mainly integrated into the nuclear genome of *C. reinhardtii* at random positions by nonhomologous end joining (NHEJ). It is proposed that the exogenous DNA is recognized, and once inserted in the genome, inactivated by chromatin condensation. This mechanism could provide protection against harmful types of DNA (Neupert et al. 2020; Einhaus et al. 2025). The strategy to increase transgene expression is to disrupt genes involved in chromatin repression. As such, the UV‐mutagenized UVM4 strain impaired in a Sir2‐type histone deacetylase (SRTA) (Neupert et al. 2009, 2020) proved to be useful in many works mentioned below, as well as the corresponding insertional mutant obtained from the CLiP library (Li et al. 2019) (ΔSRTA) (Tables 2 and 3). In accordance with these findings, the use of chemical reagents inhibiting the activity of deacetylases is effective to counteract gene silencing (Beauchemin et al. 2024). Additional epigenetic factors were recently identified, demonstrating that research on epigenetic silencing is crucial for stable transgene expression (Einhaus et al. 2025, see table 3). Another strategy to allow stable expression of transgenes is their introduction to so‐called genetic safe harbours in the genome which are not subject to strong genetic silencing (Jacobebbinghaus et al. 2025).

Other than adopting specific background strains, the design of the transgene sequences is key to allow stable expression (summarized in Perozeni and Baier 2023). The sequences must be adapted to the *C. reinhardtii* codon usage bias. The use of strong promoter and terminator couples is also fundamental. Additionally, insertion of specific introns inside the transgene sequence further improves expression. However, while this so‐called Intron Mediated Enhancement (IME) phenomenon seems widely present in *C. reinhardtii* , the underlying mechanism is not yet fully understood (Baier et al. 2020). Reported IME in *C. reinhardtii* was achieved using introns of the gene encoding the Rubisco small subunit (*RBCS2*) (Baier, Kros, et al. 2018) and a specific tool was developed to help in transgene design and intron interspersion (Intronserter, Jaeger et al. 2019). In 2018, these principles have been incorporated in the standardized Modular Cloning (MoClo) toolkit that enables the design and rapid generation of constructs using the Golden Gate Assembly technology for synthetic biology approaches (Crozet et al. 2018). Since its release, the MoClo kit was expanded with the addition of new modules and destination vectors by the community members (e.g., Mehrshahi et al. 2020; Aschern et al. 2025; Niemeyer and Schroda 2022).

### 2.2 Chloroplast Engineering

An alternative to overcome some of the limitations of nuclear transgene expression is chloroplast engineering. The chloroplast genome of *C. reinhardtii* is organized in a circular DNA of ~205 kbp present in ~83 copies/cell (Gallaher et al. 2018). Stable transgene expression is achievable due to the lack of (nuclear) silencing mechanisms. The presence of homologous recombination allows targeted gene insertion at specific loci and eliminates the issue of the positioning effect encountered in nuclear genome transformation experiments. High transgene copy number can be reached due to the multicopy nature of the chloroplast genome (Cutolo et al. 2022). Furthermore, compartmentalization can overcome possible toxicity effects of the heterologous protein expression (Cutolo et al. 2022). Limitation to chloroplast engineering in *C. reinhardtii* is mainly due to costly transformation methods (biolistics), long duration to isolate homoplasmic transgenic lines (i.e., in which the mutation/insertion is present in all copies of the plastome), restricted toolbox availability compared to the one available for nuclear engineering (Esland et al. 2018) and the possible strong proteolysis of transgenic proteins (Ma et al. 2022). Developments have been made in recent years to accelerate chloroplast engineering efforts, including systems for generation of marker‐free transplastomic lines (Taunt et al. 2023, see table 3) tuneable expression systems (Mordaka et al. 2025), as well as bidirectional promoters (Tazon et al. 2026). A collection of standard parts adapted to the Modular Cloning syntax was developed for Chlamydomonas chloroplast (Melero‐Cobo et al. 2025; Inckemann et al. 2025). In the work of Inckemann et al. (2025), the 140 standardized genetic parts were integrated into a high‐throughput platform developed to advance chloroplast engineering and rapid screening of transplastomic mutants (Inckemann et al. 2025). Furthermore, because of its compact genome and detailed information about its sequence and codon usage bias (Fages‐Lartaud et al. 2022), *C. reinhardtii* plastome is a suitable platform for synthetic redesign. Synthetic genomics can be applied to develop solutions for biological containment with the aim of preventing events of horizontal gene transfer and reducing the risks of deployment of engineered organisms especially in outdoor settings (Goold et al. 2024). In this regard, impressive work on codon reassignment and genetic code compression was performed on *C. reinhardtii* plastome (Mordaka et al. 2026).

## 3 Biotechnological Applications

Unlike *C. vulgaris* or *H. pluvialis* which present intrinsic valuable compounds (Araújo et al. 2021), *C. reinhardtii* is not remarkable in this respect. With the view of potential biotechnological applications, this is probably the reason why this microalga is extensively tested for heterologous expression of molecules of industrial interest. Research efforts are mainly focused on terpenoids and recombinant proteins of various origins. Considering terpenoids, the interest lies in the fact that the high carbon flux through the plastid‐localized methylerythritol phosphate (MEP) pathway can be diverted from the endogenous synthesis of carotenoids towards that of terpenoid precursors by expressing a sesquiterpene synthase or diterpene synthase (Figure 1). Table 2 gives a few examples of such heterologous terpenoid productions. The choice of the molecules listed in Table 2 is based on the availability of titre data (in mg/L), the comparison with titres for the production of similar molecules in other photosynthetic organisms (cyanobacteria or mosses), or the availability of titres at a large‐scale cultivation. Terpenoids are associated with unique flavours interesting for the fragrance industry (patchoulol, valencene, bisabolene, sclareol) or for their antioxidant properties in the food industry (astaxanthin). Except for astaxanthin, the extraction method is based on a dodecane monolayer addition at the top of the cultivation medium, where the terpenoid molecule is trapped and extracted. High density cultivation may require specialized photobioreactors (e.g., from the CellDEG company) and is accompanied by optimization of the medium composition (high concentration of phosphorus, CO_2_ bubbling). The highest volumetric titre of patchoulol reached in the engineered *C. reinhardtii* UPN22 strain (described in Table 3), which is able to grow on phosphite and nitrate (14.9 mg/L, Abdallah et al. 2026), is close to that obtained with *Synechocystis* sp. *PCC 6803* (17.3 mg/L, Dienst et al. 2020). In the mosses *Physcomitrella patens* and *Marchantia paleacea* , the patchoulol content reaches 1–3 mg of patchoulol/g cell dry weight (Zhan et al. 2014; Zhang et al. 2020). As a matter of comparison and given that a patchoulol titre of 0.5 mg/L in Chlamydomonas corresponds to 1 mg/g cell dry weight (Lauersen et al. 2016), the titre of 14.9 mg/L patchoulol in Chlamydomonas does correspond to 30 mg/g cell dry weight. This indicates that the Chlamydomonas carbon metabolism could be more efficiently diverted towards terpenoid biosynthesis than that of mosses. However, photosynthetic microorganisms are still behind heterotrophic chassis, such as yeast and bacteria, in which titres of 1–3 g/L are reported (Peng et al. 2023). Nootkatone, another sesquiterpene obtained by oxidation of valencene, reaches a titre of 148 mg/L in Chlamydomonas (Saudhof et al. 2026), which is similar to the titre (154 mg/L) obtained with *Synechocystis* sp. *PCC 6803* (Sun et al. 2023). In the case of bisabolene production, the titre obtained with *C. reinhardtii* is higher than the one obtained with *Synechocystis* sp. *PCC 6803* (906 mg/L versus 180 mg/L) (Sawant et al. 2024; Rodrigues and Lindberg 2021). In that specific case, the microalgal production was tested in outdoor‐natural light cultivation condition in India. Diterpene (sclareol) is another example of successful production (656 mg/L) in Chlamydomonas. By comparison, sclareol production in *Physcomitrella patens* is very low (2.38 mg/L). However, *C. reinhardtii* sclareol production is again still behind heterotrophic organisms, since engineered yeasts can reach up to 10–30 g/L of sclareol (Li et al. 2026). Astaxanthin is not naturally present in *C. reinhardtii* cells, except in a specific zygotic phase. Its synthesis has been engineered by constitutive expression of the native β‐carotene ketolase and overexpression of key and rate‐limiting enzymes of the astaxanthin biosynthesis ( *Pantoea ananatis* phytoene synthase and native β carotene hydroxylase) (Figure 1). To the best of our knowledge, the highest titre reported is 23 mg/L (Amendola et al. 2023), compared to 38.4 mg/L using *Synechococcus* sp. *PCC 11901* (Betterle et al. 2025). Titres up to 174 mg/L could be reached in the best astaxanthin producer, a UV mutagenesis mutant of the green microalga *H. pluvialis* , while 80 mg/L is the average (Hong et al. 2018). Nevertheless, the advantage of *C. reinhardtii* is linked to the easiness of cell breakage for astaxanthin extraction compared to *H. pluvialis* , in which astaxanthin is highly accumulated only in the so called ‘red phase’ reached after 20 days when cells become extremely resistant to breakage.

**FIGURE 1:**
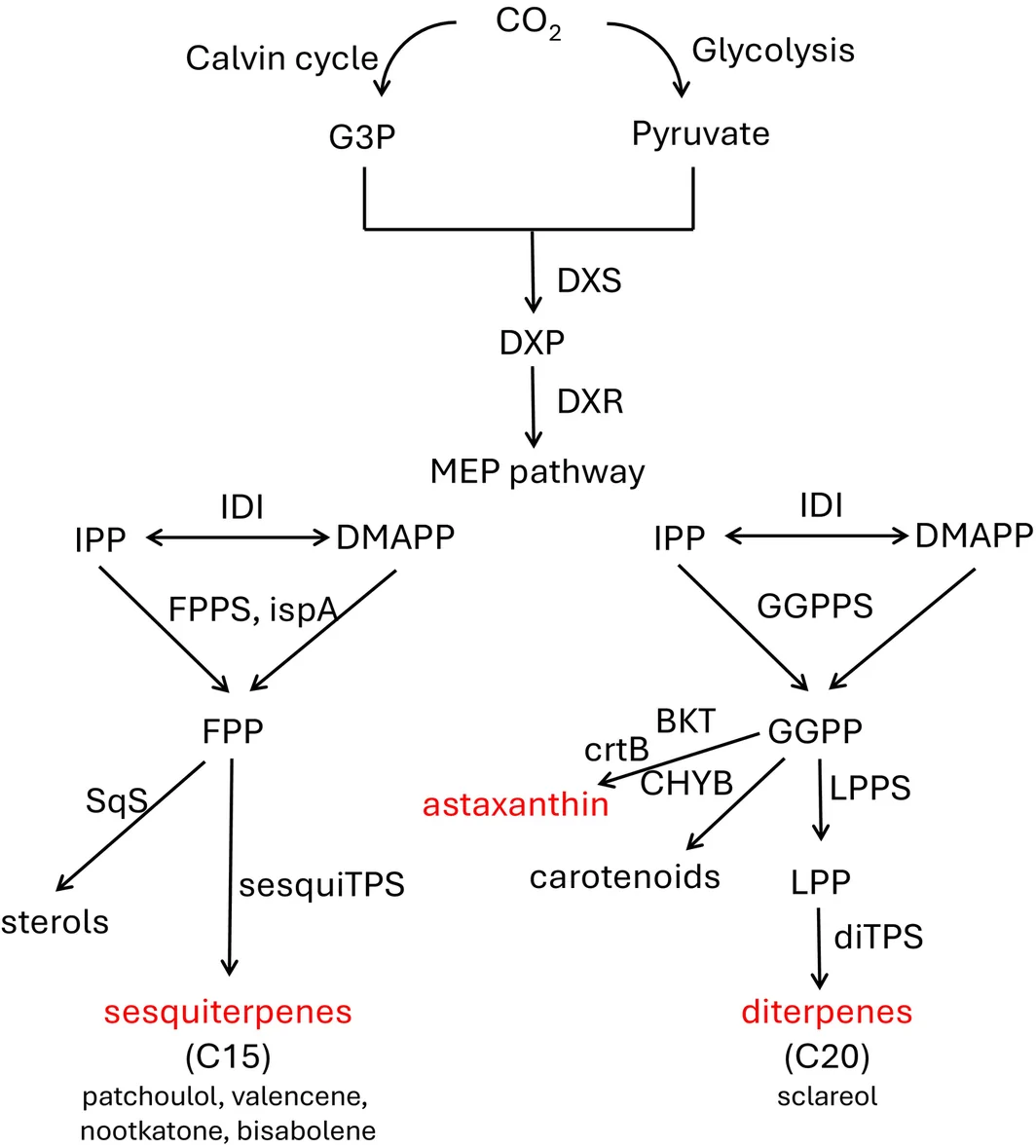
Simplified scheme of the MEP pathway with enzymes cited in Table 2. BKT, β‐carotene ketolase; CHYB, β‐carotene hydroxylase; crtB, phytoene synthase; diTPS, diterpene synthase; DMAPP, dimethyl allyl pyrophosphate; DXP, 1‐deoxy‐D‐xylulose 5‐phosphate; DXR, 1‐deoxy‐D‐xylulose 5‐phosphate reductoisomerase; DXS, 1‐deoxy‐D‐xylulose 5‐phosphate synthase; FPP, farsenylpyrophosphate; FPPS/ispA, farsenylpyrophosphate synthase; G3P, glyceraldehyde 3‐phosphate; GGPP, geranylgeranyl pyrophosphate; GGPPS, geranylgeranyl pyrophosphate synthase; IDI, isopentenyl diphosphate isomerase; IPP, isopentenyl pyrophosphate; LPP, (13E)‐8α‐hydroxylabden‐15‐yl diphosphate; LPPS, (13E)‐8α‐hydroxylabden‐15‐yl diphosphate synthase; MEP, 2‐C‐methylerythritol 4‐phosphate; sesquiTPS, sequiterpene synthase; SqS, squalene synthase.

**TABLE 2:** Research progress on expression of terpenoids and recombinant proteins in Chlamydomonas and other photosynthetic chassis.

Produced biomolecule and photosynthetic chassis	Key modifications	Growth cultivation conditions	Titre*	References
**Terpenoids**				
*Patchoulol (C15)*				
Chlamydomonas UPN22	*Pc*PS and *Sc*FPPS targeted to the chloroplast	200 mL, 6xPhi medium, 3% CO_2_ in air, CellDEG HD100 bioreactors, 14 days	14.9 mg/L	Abdallah et al. 2026
*Synechocystis* sp. PCC 6803	*Pc*PS	10 mL, bicarbonate as CO_2_ source, CellDEG bioreactors, 8 days	17.3 mg/L	Dienst et al. 2020
*Physcomitrella patens*	*Pc*PS	PhyB medium, 14 days	1.3 mg/g CDW	Zhan et al. 2014
*Marchantia paleacea*	*Pc*PS	1/2 Gamborg agar medium, 4 weeks	3.3 mg/g CDW	Zhang et al. 2020
*Valencene (C15)*				
UVM4	*Cn*VS‐YFP‐ispA	4.5 mL, Tris‐acetate‐phosphate medium, 6‐well microtiter plate, 6 days	11.1 mg/L	Saudhof et al. 2026
*Synechocystis* sp. PCC 6803	*Cn*VS *Sc*IDI‐ispA‐*Ec*DXS	100 mL, semi‐continuous, 3% CO_2_ in air, column photobioreactor, 36 days	154 mg/L	Sun et al. 2023
*Nootkatone (C15)*				
UVM4	*Cn*VS‐YFP‐ispA, HPO‐RFP and *Sp*DXS‐CFP targeted to the chloroplast	2.5 L, 6xP medium, 3% CO_2_ in air, CellDEG HD1500, 15 days	148 mg/L	Saudhof et al. 2026
*Bisabolene (C15)*				
UVM4	3×*Ag*Bs, SQs ↓	5 L tubular reactor, optimized HSM medium, 10 g/L bicarbonate supplementation, 6 days	906 mg/L	Sawant et al. 2024
*Synechocystis* sp. PCC 6803	*Cf*DXS—sIDI‐*Ag*Bs‐ispA	8 mL, CellDEG bioreactor, 10 days	180 mg/L	Rodrigues and Lindberg 2021
*Sclareol (C20)*				
UVM4	*Sp*DXS2, *Ss*SCS, *Ss*LPPS targeted to the chloroplast	2.5 L, CellDEG HD1500 bioreactor, 3% CO_2_ in air, 19 days	656 mg/L	Einhaus et al. 2022
*Physcomitrella patens*	*Ss*LPPS—*Ss*SCS	Agar PhyB medium, 18 days	2.28 mg/L	Pan et al. 2015
*Astaxanthin (C40)*				
UVM4	*Cr*BKT, *Pa*crtB, *Cr*CHYB targeted to the chloroplast	80 mL, 6xP medium, 3% CO_2_ in air, Multi‐Cultivator MC‐1000, 4 days	23.3 mg/L	Amendola et al. 2023
*Synechococcus* sp. *PCC 11901*	*C*rBKT, crtZ	80 mL, Multi‐Cultivator MC‐1000, 3% CO_2_ in air, 4 days	38.4 mg/L	Betterle et al. 2025
*Haemetacoccus pluvialis* mutant M13	UV mutagenesis and selection of astaxanthin high producers	5 L, 10 days vegetative growth, 20 days for induction of the red stage	174 mg/L	Hong et al. 2018
**Recombinant proteins**				
*SARS‐CoV‐2 spike protein*				
CC‐4533 (ΔSRTA)	N‐terminal portion of the SARS‐CoV‐2 spike protein, ARS2 secretion signal	TAP medium	11.2 μg/L	Kiefer et al. 2022
*Zeolin*				
UVM4	Maize gamma‐zein and bean phaseolin	TAP medium	82 μg/L	Perozeni et al. 2023
*Lolium perenne* *ice‐binding protein*				
UVM4	*g*Luc or *c*CA secretion signals	10 L, TAP medium, semi‐continuous	10 mg/L	Lauersen et al. 2013
*Human epidermal growth factor (hEGF)*				
UVM4	FEA2 secretion signal	TAP medium, 2 days	75–100 μg/L	Baier, Wichmann, et al. 2018
*Tyrosine N‐monooxygenase of* *Sorghum bicolor*				
TN72	Cloned and expressed from the chloroplast genome	120 L, TAP medium, 0.5% CO_2_ in air, 7 days	6.77 mg/L	Zedler et al. 2016
*Diterpene synthase of* *Abies balsamea*				
TN72	Cloned and expressed from the chloroplast genome	120 L, TAP medium, 0.5% CO_2_ in air, 7 days	22 mg/L	Zedler et al. 2016
*Mammary‐associated serum amyloid A*				
Scene	Cloned and expressed from the chloroplast genome	100 L, HSM, 5% CO_2_ in air, 12 days	3.28 mg/L	Gimpel et al. 2015

*Note:* Strains: UVM4: UV‐mutagenized strain impaired in a Sir2 histone deacetylase (STRA); UPN22: derivative of UVM4 able to grow on phosphite and nitrate, CC‐4533: insertional mutant inactivated for SRTA (ΔSRTA); TN72: cell wall less strain (cw15) where the resistance cassette to spectinomycin (aadA) is located in the psbH subunit of photosytem II (psbH::aadA); Scene: *C. reinhardtii* strain expressing *Scenedesmus obliquus* psbA‐m‐SAA (mammary‐associated serum amyloid A).

Abbreviations: AgBs, *Abies grandis* bisabolene synthase gene; ARS2, arysulfatase 2 of *C. reinhardtii* ; *c*CA, secretion signal peptide of carbonic anhydrase 1 protein of *C. reinhardtii* ; CfDXS, *Coleus forskohlii* 1‐deoxy‐D‐xylulose‐5‐phosphate synthase; CnVO, *Callitropsis nootkatensis* valencene oxidase; CnVS‐YFP‐ispA, *Callitropsis nootkatensis* valence synthase –*mVenus* fluorescent protein‐ *E. coli* farsenylpyrophosphate synthase; CrBKT, *C. reinhardtii* β‐carotene ketolase; CrCHYB, *C. reinhardtii* β‐carotene hydroxylase; crtZ, *Brevundimonas* sp. *SD‐2*12 β‐carotene hydroxylase; EcDXS, *E. coli* 1‐deoxy‐D‐xylulose‐5‐phosphate synthase; FEA2, iron assimilation 2 protein of *C. reinhardtii* ; HPO‐RFP, *Hyoscyamus muticus* premnaspirodiene oxygenase—mRuby2 Fluorescence protein; HSM, high salted medium; *g*Luc, secretion signal peptide of *Gaussia princeps* luciferase; P, phosphate. PacrtB, *Pantoea ananatis* phytoene synthase; PcPS, *Pogostemon cablin* patchoulol synthase; Phi, phosphite; ScFPPS, *Saccharomyces cerevisiae* farsenylpyrophosphate synthase (ERG20); ScIDI, *S. cerevisiae* isopentenyl diphosphate isomerase; sIDI, *Synechocystis* sp. *PCC 6803* IPP/DMAPP isomerase; SpDXS2, *Salvia pomifera* 1‐deoxy‐D‐xylulose‐5‐phosphate synthase; SpDXS‐CFP, *Salvia pomifera* 1‐deoxy‐D‐xylulose‐5‐phosphate synthase—*‐* *mCerulean* Fluorescent protein; SQs, knock down of squalene synthase; SsLPPS, *S. sclarea* (13E)‐8α‐hydroxylabden‐15‐yl diphosphate synthase; SsSCS, *Salvia sclarea* sclareol synthase; TAP, tris‐acetate‐phosphate medium.

* Highest titres obtained using growth in the light at the end of the cultivation.

In conclusion, even if the conditions of production are not exactly equivalent, the results presented in Table 2 indicate that cyanobacteria (*Synechocystis* and *Synechococcus*) and *C. reinhardtii* are roughly comparable in terms of terpene bioproduction. In some cases, production in *C. reinhardtii* is even better, or at least, larger volumes were tested, which is favourable for future upscaling production.

Considering therapeutics, *C. reinhardtii* has additional advantages over the sustainability aspect. Indeed, *C. reinhardtii* possesses the ‘Generally Recognized as Safe’ (GRAS) status from the USA Food and Drug Administration (FDA) and is approved as a new food ingredient from the National Health Commission of the People's Republic of China (Zhou et al. 2025). This means that using it as host for therapeutics production could reduce the purification cost of the recombinant proteins by direct use through topic applications or oral vaccines, especially for animals for which other delivery strategies can be impractical (Dyo and Purton 2018). Furthermore, the chloroplast offers a suitable compartment for expression of complex biomolecules, including proteins, that could be toxic in other traditionally used eukaryotic hosts (e.g., yeast and mammalian cells), for examples immunotoxins, which find application in cancer treatment (Dyo and Purton 2018). However, a critical aspect to consider for commercialization of recombinant proteins as therapeutics is the characterization of the post‐translational modifications to avoid possible immunogenic reactions. Recent work has been initiated on this matter to investigate the glycosylation pathway of *C. reinhardtii* with the perspective to adapt it to produce human‐compatible biological products (Leprovost et al. 2024). Table 2 gives some examples for which titres (mg/L) are available. The titres are variable, ranging from 11.2 μg/L for the SARS‐CoV‐2 spike protein (Kiefer et al. 2022) to 22 mg/L for the diterpene synthase of *Abies balsamea* (Zedler et al. 2016). The titres are in general lower than those obtained for the terpenoid production for which some titres reach levels above 100 mg/L, as discussed previously. Secretion signals can be added to facilitate the recovery of the recombinant proteins (SARS‐CoV‐2 spike protein, *Lolium perenne* ice‐binding protein, human epidermal growth factor). In case of proteins expressed from the chloroplast genome (diterpene synthase, monooxygenase, mammary‐associated serum amyloid A), production was tested at large scale (100–120 L) (Zedler et al. 2016; Gimpel et al. 2015) and for some of them at facilities of a private company (A4F, Portugal). This demonstrates that *C. reinhardtii* may represent an attractive platform to produce recombinant proteins, although these trials were not translated at the industrial level.

## 4 Development of Chlamydomonas Super Strains for Industrial Biotechnology

The extensive genetic toolbox and resources available for Chlamydomonas manipulation provide all the necessary means for the development of highly engineered strains adapted for industrial production. The main areas of improvement are the following: increasing the resistance to both biotic (e.g., pathogens) and abiotic (e.g., low/high light) stresses, enhancing biomolecule accumulation as well as high and stable transgene expression (Benedetti et al. 2018). Over the years many different strategies have been tested to improve Chlamydomonas traits in one or more of these areas (Benedetti et al. 2018; Kumar et al. 2020). A selection of recent and relevant works on the matter is critically discussed hereafter (Table 3).

**TABLE 3:** Selected recent engineering efforts to improve the biotechnological traits (cultivation and biomolecule production) of *Chlamydomonas reinhardtii* .

Objective	Strategy	Recipient strain*	Selection system	Maximum scale tested	Key outcomes	References
Improve nuclear transgene expression levels and stability over time	CRISPR‐Cas9 targeted KO of 11 putative epigenetic factors involved in transgene silencing (e.g., SRTA)	CC‐4350	Hygromycin^R^ (initial single KO), ARG7 (second KO) and Nourseothricin^R^ (third)/ARG7 (first KO) and Split Spectinomycin^R^ (second and third)	50 mL (flasks)	27 new strains/high and stable transgene expression tested after 3 months	Einhaus et al. 2025
Expand trophic flexibility	OE of *At*STP1 transporter	CC‐125	Hygromycin^R^	50 mL (flasks)	Effective glucose uptake/glucose‐to‐biomass conversion yield of ~19%	Shin et al. 2026
Reduce contamination impact in high cell density cultures	Combined chloroplast PtxD expression and nuclear NIT1‐2 expression	UVM4	Growth in Phosphite and Nitrate	200 mL (in CellDeg HD100 bioreactor)	UPN22 strain and phosphite buffered media validation	Abdallah et al. 2022
Boost lipid production	CRISPR‐Cas9 KO of *Cr*CTI1	CC‐4349	Hygromycin^R^	N/A	~5‐fold increase in TAGs (up to ~0.4 μg 10^6^ cells^−1^) with no impact on cell growth in mixotrophic conditions	Li et al. 2025
Enhance lipid content	OE of MYB1 transcription factor	UVM4	Paromomycin^R^	N/A	1.9 to 3.2‐fold increase in TAGs (up to ~0.2 μg 10^6^ cells^−1^) in mixotrophic conditions/increase in starch and protein content (up to ~10 and ~25 μg 10^6^ cells^−1^)	Zhao et al. 2023
Improve carbon fixation	OE of a cyanobacterial FBP/SBPase	UVM4	Paromomycin^R^	80 mL (in Multi‐Cultivator MC 1000‐OD)	Increased cell volume and ~60% higher sedimentation rate	Bussola et al. 2025
Reduce self‐shading in high density cultures	CRISPR‐Cas9 KO of LYCE and ZEP**	CC‐4349	Hygromycin^R^	80 mL (flasks)	60% lower Chl content/Improved growth in high light phototrophic conditions (reaching ~35 10^6^ cells mL^−1^ at 5% CO_2_ bubbling)	Kim et al. 2024
Develop a marker‐free system for chloroplast engineering	HR to disrupt *psaA‐3* gene	CC‐1690	Spectinomycin^R^ ***	50 mL (flasks)	HNT6 strain validation	Taunt et al. 2023

Abbreviations: ARG7, argininosuccinate lyase; *At*STP1, sugar transport protein 1 (STP1) from *Arabidopsis thaliana* ; Chl, chlorophyll; CTI1, carboxyltransferase interactor 1; FBP/SBPase, fructose‐1,6−/sedoheptulose‐1,7‐bisphosphatase; HR, homologous recombination; KO, knock‐out; LCYE, lycopene ε‐cyclase; MYB1, R2R3‐type MYB family transcription factor; N/A, not applicable; NIT1, nitrate reductase (also called NIA1); NIT2, transcription factor regulating nitrogen metabolism; OE, over‐expression; *psaA‐3*, photosystem I P700 chlorophyll a apoprotein A1 exon 3; PtxD, phosphite oxidoreductase from *Pseudomonas stutzeri* WM88; TAGs, triacylglycerols; ZEP, zeaxanthin epoxidase.

* Description of the strains is available on https://chlamycollection.org.

** The mutant was generated in a previous study (Song et al. 2020).

*** The antibiotic cassette is removed by integration of the transgene by HR.

As previously discussed, the UVM4 strain is largely adopted as a recipient chassis to allow stable transgene expression. In Einhaus et al. (2025), new candidates involved in nuclear transgene silencing were identified and the number of biotechnologically relevant strains for stable transgene expression was expanded by achieving CRISPR‐Cas9‐targeted single, double and triple knockout mutants (Einhaus et al. 2025). Of all generated strains, the triple‐KO ΔA51 mutant (i.e., ΔSRTA, ΔDMC5 and ΔVIG1), combining the disruption of the following genes/functions: *SRTA*(*HDA17*)/Sir2‐type histone deacetylase, *DMC5*(*MET1*)/DNMT1‐type cytosine‐C5 DNA methylase and *VIG1/*Ortholog of the Vasa intronic gene, showed the highest transgene expression abilities (Einhaus et al. 2025). Furthermore, the triple mutant maintained high transgene expression levels after 3 months supporting long‐term stability against silencing. By contrast, both UVM4 and ΔSRTA single KO strains showed decreased level of expression (−16% and −10%, respectively) in the long term. Authors hypothesize that *SRTA* deficiency alone protects only at an early stage, while other factors might be involved in the epigenetic silencing after mitosis and cell proliferation.

Expanding the trophic flexibility of Chlamydomonas is a highly desirable trait to implement for industrial applications. Recently, Shin et al. overexpressed the Sugar Transport Protein 1 (STP1) from *A. thaliana* in Chlamydomonas and compared the achieved heterotrophic productivity with transformants expressing previously published transporters (Shin et al. 2026). In dark conditions, the *At*STP1 expressing strain exhibited the highest biomass production (0.325 g/L DCW) and highest glucose‐to‐biomass conversion yield (of ~19%). However, the growth levels are still unsatisfactory when compared to the heterotrophic growth in acetate‐containing media (Shin et al. 2026). Alternatively, a non‐canonical substrate to sustain Chlamydomonas growth which showed a relevant industrial potential is inorganic phosphite. Indeed, this phosphorus source cannot be metabolized by many competing organisms such as most bacteria, plants and fungi, therefore representing a promising solution for reducing contamination risk especially in the context of heterotrophic growth conditions or cultivation in open ponds (Benedetti et al. 2018). Chlamydomonas was shown to metabolize this substrate upon expression of the phosphite oxidoreductase PtxD from *Pseudomonas stutzeri* WM88 either from the nuclear (Loera‐Quezada et al. 2016) or the chloroplastic genomes (Changko et al. 2020). Building on the previous works, the UVM4 chassis was used to deploy a combinatorial engineering approach aiming at enhancing its metabolism for high cell density growth (Abdallah et al. 2022). For this strategy, a new growth solution was prepared, containing phosphite and nitrate. Although nitrate is a less preferred nitrogen source for Chlamydomonas than ammonium, its use finds application in large‐scale cultivation setups since its consumption does not lead to important pH changes in the media. Because many laboratory strains, including UVM4, have lost the ability to grow on nitrate, UVM4 was engineered to restore the ability to use nitrate by expressing *NIT1‐2* genes from the nuclear genome and to promote phosphite metabolization through the chloroplast expression of PtxD. The resulting strain was further engineered for the heterologous production of terpenoids. When tested in non‐axenic conditions, it was able to grow to high cell density (> 10^7^ cells/mL) and to produce ∼6.2 mg/L of patchoulol without being impacted by yeast contamination.

While Chlamydomonas is not an oleaginous microalga, the biosynthesis of these biomolecules can be enhanced under stress conditions, such as growth under nitrogen starvation (Zhao et al. 2023). However, recent works demonstrated that it is possible to improve Chlamydomonas lipid metabolism without compromising cell growth or affecting its photosynthetic performances. In the work from Li and collaborators, the Chlamydomonas homologue of the plant carboxyltransferase interactor 1 (CrCTI1) was identified as a negative regulator of fatty acid synthesis (Li et al. 2025). By knocking out this enzyme via CRISPR‐Cas9 technology, they engineered strains capable of accumulating up to five times more triacylglycerols (TAGs) stored in lipid droplets in mixotrophic conditions. Similarly, Zhao et al. boosted lipid production in standard conditions by overexpressing the MYB1 transcription factor, a positive regulator of lipid production under N deprivation (Zhao et al. 2023). While the resulting strains accumulated lower levels of TAGs compared to the CrCTI1 KO mutants, they showed a parallel increase in starch content and in biomass production.

To enhance productivity and the light‐to‐biomass conversion efficiency, many strategies focused on tackling the bottlenecks of carbon fixation and regulating light use efficiency (Benedetti et al. 2018; Kumar et al. 2020). Overexpression of the *Synechococcus elongatus* fructose‐1,6−/sedoheptulose‐1,7‐bisphosphatase (FBP/SBPase) dual enzyme in *C. reinhardtii* UVM4 strain led to an increased growth rate in mixotrophic conditions, as well as increased cell volume in both mixotrophic and phototrophic conditions (Bussola et al. 2025). The increase in cell dimensions was accompanied by around a 2‐fold increase in starch and protein content as well as a 1.5‐fold increase in lipid content, on a cell basis. Lastly, a ~60% higher sedimentation rate was observed in the FBP/SBPase expressing strains compared to the UVM4 reference strain, which is biotechnologically relevant because it could help improve the downstream process of cell harvesting. On the other hand, Kim and collaborators showed that the *Δzl* CRISPR Cas9 double knockout mutant strain for the lycopene ε‐cyclase (LCYE) and zeaxanthin epoxidase (ZEP) genes had significantly better growth performances in high‐light phototrophic conditions compared to the parental strain (Kim et al. 2024). This improvement is probably linked to a lower chlorophyll content of the *Δzl* strain, allowing for better light penetration and counteracting the effects of self‐shading typically present in high cell density cultures (Kim et al. 2024).

Even if these (and other) engineering efforts show us a promising way towards the domestication of Chlamydomonas for biotechnological purposes, there are still important limitations that need to be addressed. Most of these strategies require the use of antibiotic selection for the isolation of mutants and the expression of the proteins of interest, which increases safety issues relative, for instance, to possible transmission of the resistance to other microorganisms. This is why the development of marker‐free systems for the generation of transgenic Chlamydomonas strains is essential. For example, the number of chassis adapted to chloroplast engineering was increased by isolation of the HNT6 recipient line (Taunt et al. 2023). This line is a photosystem I mutant inactivated in the 3rd exon of *psaA* by an antibiotic resistance cassette. The psaA functionality is restored by removal of the antibiotic cassette and simultaneous integration of the transgene by homologous recombination. Transformants are selected in high light for restoration of phototrophy. Additionally, HNT6 was generated from a wild type walled strain to potentially overcome the shortcoming observed in large scale cultivation of TN72 (mentioned in Table 2), which is cell wall less and was shown to be more sensitive to shear stress and thus requiring a more complex downstream processing (Taunt et al. 2023; Stoffels et al. 2019). Ideally, one would need to combine several of these traits for biotechnological applications. For nuclear targeting, the long‐term stability of transgene expression is essential. The possibility to reach high density cultures is also desirable, possibly by decreasing the chlorophyll content while avoiding contamination by using phosphite‐dependent strains and stabilizing the pH using nitrate as nitrogen source. For biorefinery purpose, the addition of a marker leading to higher lipid content/TAG accumulation could be interesting for increased valorization and circularity purpose.

Biofoundries are being built all over the world to accelerate chassis engineering by providing automated and integrated infrastructures based on the Design‐Build‐Test‐Learn (DBTL) principles of synthetic biology (Hillson et al. 2019). These platforms are characterized by robotics‐driven automation, which guarantees high reproducibility, as well as by high throughput analytics and workflows, and they therefore represent a solution to bridge the gap between laboratory and industrial scale applications. In this regard, the feasibility of such projects for photosynthetic chassis is demonstrated by the Paris Biofoundry (www.parisbiofoundry.org), actively developing new technology for microalgae (e.g., *C. reinhardtii* ) and cyanobacteria (Douchi et al. 2026).

## 5 Conclusions and Perspectives

Research data presented above demonstrate that *C. reinhardtii* is a photosynthetic chassis with dedicated synthetic biology toolkits and platforms where investments towards industrial applications are still active as shown by the recently established biofoundry in Paris. However, to the best of our knowledge, the production of biomolecules, although very diverse as shown above, remains at laboratory/pilot‐scale levels and commercialization of a product from this microalga is still awaited. Possible reasons are the overall production costs, the low level of automation of operations, the scarcity of experienced staff in microalgae industrial biotechnology, and the use of highly genetically engineered strains (reviewed in Novoveská et al. 2023). These problems are not restricted to *C. reinhardtii* but to all phototrophic cultivations (Novoveská et al. 2023). Considering the European Union for instance, microalgal‐based biotech companies rely on few species: mainly *Chlorella* spp., *Nannochloropsis* spp., and *H. pluvialis* (Araújo et al. 2021), which are not genetically manipulated. One reason is that the adoption of new species and especially highly engineered strains is frequently hindered by expensive and time‐consuming administrative processes (Araújo et al. 2021). Precautionary risk assessments are necessary to evaluate the biosafety of genetically modified microalgae (GMM) on human health as well as animals and environmental protection and international harmonized regulations are still lacking (Onn et al. 2025). Researchers hope that the development of regulatory frameworks will balance the precautionary principle with the benefits of GMM applications, especially in the case of genome editing technologies (Spicer and Molnar 2018). To this end, clear communication from the (research) institutions to the public of the risks and benefits of GMM is important to improve social acceptance (Calatrava et al. 2024).

## Author Contributions

**Sofia Inturri:** writing – original draft, writing – review and editing, visualization. **Nicolas Rouhier:** writing – review and editing, funding acquisition, supervision. **Claire Remacle:** writing – review and editing, funding acquisition, supervision, writing – original draft, visualization.

## Funding

This work was supported by Fonds National de la Recherche Scientifique, CDR J.0149.23 (Belgium), CDR J.079.26 (Belgium) and Université de Lorraine.

## Conflicts of Interest

The authors declare no conflicts of interest.

## Data Availability

Data sharing not applicable to this article as no datasets were generated or analysed during the current study.
